# Concurrent intraductal papilloma and nipple adenoma: a case report and review of the literature

**DOI:** 10.1093/jscr/rjag381

**Published:** 2026-05-24

**Authors:** Angelo Federico, Sara Glendinning, Helen Mabry

**Affiliations:** General Surgery, Corewell Health, Dearborn, MI 48124, United States; General Surgery, Corewell Health, Dearborn, MI 48124, United States; Breast Surgical Oncology, Mercy Health, Perrysburg, OH 43551, United States

**Keywords:** nipple adenoma, intraductal papilloma, histopathology, malignancy

## Abstract

Nipple adenoma is a rare benign proliferative lesion that makes up <1% of diagnosed benign breast lesions. The diagnosis of an intraductal papilloma is more common and represents an estimated 2%–3% of all benign breast tumours. The concomitant diagnoses of these pathologies together is extremely rare. Here, we report the case of a 34-year-old female that presented with a left-sided nipple lesion discovered on self-exam. Subsequent imaging was classified as BIRADS 4A and the patient opted to undergo surgical excision. Resultant pathology revealed both nipple duct adenoma and intraductal papilloma with florid ductal hyperplasia and focal atypia. Given the rarity of underlying nipple pathologies and the associated diagnostic difficulties, a thorough and complete workup is crucial even when benign breast pathology is suspected.

## Introduction

Nipple adenoma is a rare but well documented benign proliferative lesion that makes up <1% of diagnosed benign breast lesions with an incidence highest in women in their fourth decade of life [1]. The diagnosis of a nipple adenoma can be difficult to make and often requires surgical excision with formal histologic evaluation prior to complete diagnosis given that it can mimic various other pathologies [2]. The diagnosis of an intraductal papilloma, however, is more common with a highest incidence in women in their third through fifth decade of life and represents an estimated 2%–3% of all benign breast tumours [3]. The concomitant diagnoses of these pathologies together is extremely rare and the available literature regarding a simultaneous diagnosis is very limited. The appropriate workup of nipple lesions is imperative to ensure the correct diagnosis is made to allow for the appropriate treatment and to ensure concomitant pathologies are not missed.

Here, we report a case of a patient with biopsy proven concomitant nipple adenoma and intraductal papilloma. This article aims to emphasize the importance of a complete workup when benign breast pathology is suspected given the possibility of underlying pathologies and to explore the associated diagnostic difficulties.

## Case report

**Figure 1:**
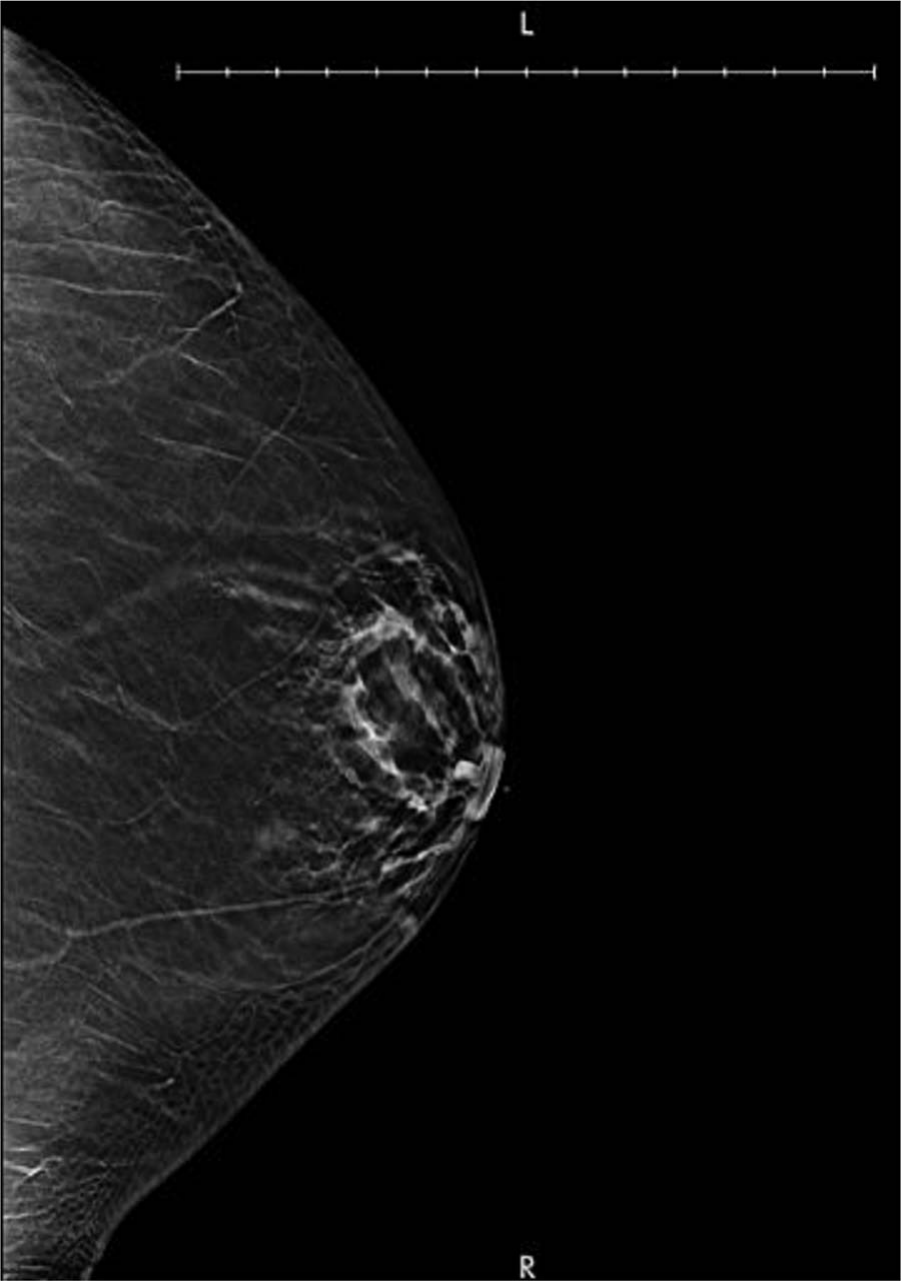
Diagnostic mammogram demonstrating an asymmetric prominence of the left nipple with a BI-RADS classification of 4A with low suspicion for malignancy.

**Figure 2:**
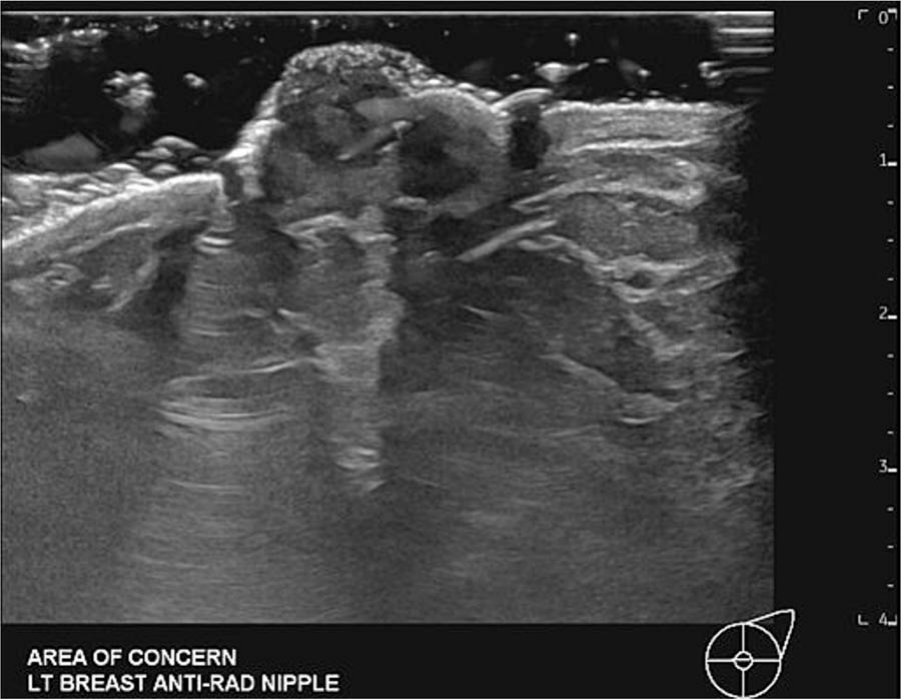
Targeted diagnostic left breast ultrasound demonstrating an enlarged left nipple and heterogenous with diffuse asymmetrically increased vascularity.

A 34-year-old female presented to the breast clinic with concerns of a left-sided nipple lesion that was discovered on self-exam. The patient states she noticed the lesion two years prior and endorsed a steady increase in size over this time. On physical examination, the lesion was noted to be flesh colored, located in the center of the patient’s nipple, and measured 1.1 cm in size. No lymphadenopathy, skin dimpling, or additional overlying skin changes were appreciated. She subsequently underwent a diagnostic mammogram which revealed a fibroglandular density and asymmetric prominence of the left nipple with a BIRADS 4A classification with low suspicion for malignancy (Fig. 1). Ultrasound was then performed and demonstrated a 9 mm complex fluid collection within the nipple which appeared benign in nature (Fig. 2). Given the desire for an accurate diagnosis and cosmetic concerns of the patient, she opted for surgical excision of the lesion. Under intravenous sedation, a circumferential incision was made around the palpable mass and it was subsequently dissected free from the surrounding breast tissue using sharp dissection. Once removed, the specimen was oriented and prepared for pathology. The wound was then examined and no residual nipple abnormalities were noted on visual inspection or manual palpation. Hemostasis was achieved via electrocautery and the wound was closed primarily. The resultant pathology revealed both nipple duct adenoma and intraductal papilloma with florid ductal hyperplasia and focal atypia, without evidence of malignancy. The patient did well postoperatively with satisfactory cosmetic results.

## Discussion

Nipple adenoma is a rare benign proliferative lesion that originates from the lactiferous ducts and is commonly described as a well-demarcated, crusting erosion within the nipple that can mimic malignant breast lesions, eczema, or Paget’s disease [4, 5]. Given the potential variability in presentation, the diagnosis of nipple adenoma can be difficult to make leading to diagnostic delay or inaccurate diagnosis. Mammography is typically part of the diagnostic pathway, but may be normal or non-diagnostic in certain cases. Ultrasound can be useful in the workup as well with typical features including a hypoechoic mass and possibly internal vascularity within or beneath the nipple [6]. Surgical excision with formal histologic evaluation is required to make a diagnosis given that the physical exam can be non-specific and imaging may be non-diagnostic [2].

In comparison, intraductal papilloma is more common than nipple adenoma with a higher estimated overall incidence of 2%–3% of benign breast tumors [3]. The most characteristic associated symptom is bloody nipple discharge. Mammography often demonstrates a well-defined small mass, dilated ducts, or small calcifications, but can be normal on occasion [7]. Ultrasound findings typically include a small hypoechoic nodule with a dilated breast duct [8]. Diagnosis typically includes core needle biopsy to determine the presence of atypia and possible need for surgical excision [7].

The concomitant diagnoses of these pathologies together is extremely rare and the available literature regarding a simultaneous diagnosis is very limited. A thorough workup of nipple lesions is imperative to ensure the correct diagnosis is made to allow for the appropriate treatment and to ensure concomitant pathologies are not missed. A complete workup includes performing a thorough history and physical exam, relevant imaging, and ultimately surgical excision. In our case, the patient underwent both mammography and ultrasound imaging; neither of these modalities demonstrated radiographic evidence of an intraductal papilloma. In addition, she did not have any pathologic symptomatology, such as bloody nipple discharge. This reinforces the need for surgical excision to both confirm the suspected diagnosis, as well as rule out any potentially existing underlying pathologies. If patients choose to pursue less invasive diagnostic approaches, the possibility of underlying pathology must be discussed in detail to allow for informed decision making from the patient given the risk of synchronous disease. Our case described above highlights the rare occurrence of concomitant nipple adenoma and intraductal papilloma, as well as brings much needed attention to the importance of a complete workup even when benign breast pathology is suspected.

## Conclusion

This case specifically highlights the complexity of diagnosing concomitant breast pathologies. While the available literature regarding the simultaneous diagnosis of intraductal papilloma and nipple adenoma is extremely limited, this case reinforces the need for a complete workup to ensure that no underlying pathology, whether benign or malignant, is missed and that the appropriate treatment is performed to improve clinical outcomes.
